# The fitness costs of reproductive specialization scale inversely with organismal size

**DOI:** 10.1073/pnas.2536055123

**Published:** 2026-04-06

**Authors:** Christopher Zhang, Eric Libby, Anthony Burnetti, Matthew D. Herron, William C. Ratcliff

**Affiliations:** ^a^School of Biological Sciences, Georgia Institute of Technology, Atlanta, GA 30332; ^b^Interdisciplinary Graduate Program in Quantitative Biosciences, Georgia Institute of Technology, Atlanta, GA 30332; ^c^Department of Mathematics and Mathematical Statistics, Integrated Science Lab, Umeå Centre for Microbial Research, Umeå University, Umeå 90187, Sweden; ^d^National Science Foundation, Alexandria, VA 22314

**Keywords:** multicellular evolution, germ-soma differentiation, reproductive specialization, scaling laws

## Abstract

The evolution of reproductive specialization, in which somatic cells forfeit reproduction, represents a fundamental innovation in complex multicellular life. This specialization imposes a fitness cost: because somatic cells do not produce offspring, organisms that invest in soma have reduced fecundity. The magnitude of this cost might be expected to depend simply on the proportion of cells allocated to soma. Here, we show that these costs also decrease with the logarithm of organism size, because larger organisms require proportionally more cell divisions for development, diluting the rate at which reproductive costs compound across multicellular generations. We derive this result analytically and validate it with data from the volvocine green algae. When somatic cells provide a compensating survival benefit, a positive feedback emerges: larger organisms can afford greater somatic investment, which in turn favors further size increases. This size-scaling relationship helps explain the broad association between large organism size and multicellular complexity.

## Results and Discussion

Germ-soma differentiation is one of the most consequential innovations in the evolution of complex life (1). The separation of reproductive and somatic functions enabled the morphological diversity characterizing “complex” multicellular organisms, yet this innovation imposes a fundamental cost: somatic cells, by definition, forfeit reproduction to perform other functions.

Some evolutionary innovations impose costs on exponential growth rates while providing compensating benefits. The twofold cost of males is the canonical example (2): sexual populations grow at half the rate of asexual populations because males cannot directly generate offspring, requiring substantial benefits to maintain sex (3). This illustrates a general principle: maximum growth rates are achieved when replicators produce more replicators, and any investment in nonreproductive functions requires sufficient compensating benefits to be evolutionarily stable. We apply this framework to understand the evolution of reproductive specialization, often referred to as germ-soma differentiation. In multicellular organisms with specialized cell types, somatic cells provide novel functionality but are not eligible to create propagules, and are discarded at reproduction. The severity of this reproductive cost should constrain which organisms can afford extensive cellular differentiation.

Empirically, there appears to be a broad connection between organism size and investment in somatic cells. Larger multicellular organisms have evolved a greater number of somatic cell types (4), but we lack systematic data on how the proportion of cells allocated to soma scales with organism size. The volvocine green algae provide insight into the role of organism size in somatic investment: cellular differentiation is absent in small species like *Gonium* and *Pandorina*, while larger species like the ∼2,048-celled *Volvox carteri* contain less than 1% germ cells (5). Why do larger multicellular algae evolve more somatic investment? In the case of the volvocine green algae, it is complicated: there are both size-dependent benefits to specialization and lower fitness costs of somatic investment at larger size (6).

In this paper, we demonstrate that the effect of organism size on the costs of reproductive specialization is not specific to the volvocine algae, but is a general mathematical consequence of clonal development from unicellular propagules, suggesting it has shaped the evolution of reproductive specialization across independently evolved multicellular lineages.

**Fig. 1.**
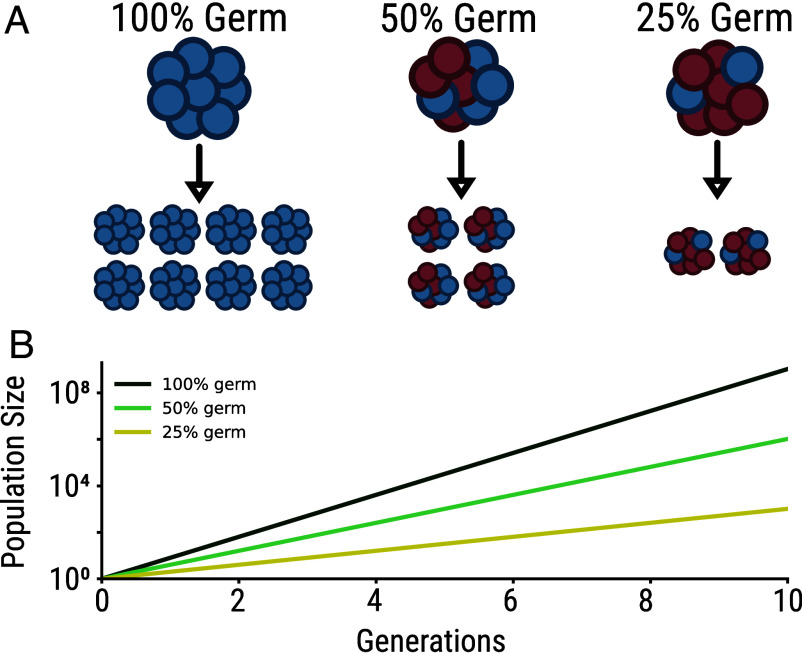
Germ-soma differentiation reduces maximum population growth rates. (*A*) Schematic showing multicellular organisms with different proportions of germ cells (blue) and somatic cells (red). At reproduction, only germ cells contribute to the next generation, while somatic cells are discarded. (*B*) Population growth trajectories over 10 generations for organisms with 100% germ cells (black), 50% germ cells (light green), and 25% germ cells (yellow).

To show this, we develop a simple model of multicellular life cycles, focusing on organisms that develop clonally from single cells, then release germ cells to initiate the next generation. Only germ cells contribute to the next generation, while somatic cells are discarded at reproduction, creating an inherent cost: organisms with more somatic cells produce fewer offspring, reducing their population growth rate in the same way that males reduce the growth rate of sexual populations (Fig. 1). Our model reveals that while this cost is substantial for small organisms, it decreases logarithmically with organism size, with larger organisms experiencing proportionally smaller reductions in growth rate for the same investment in somatic cells.

**Fig. 2.**
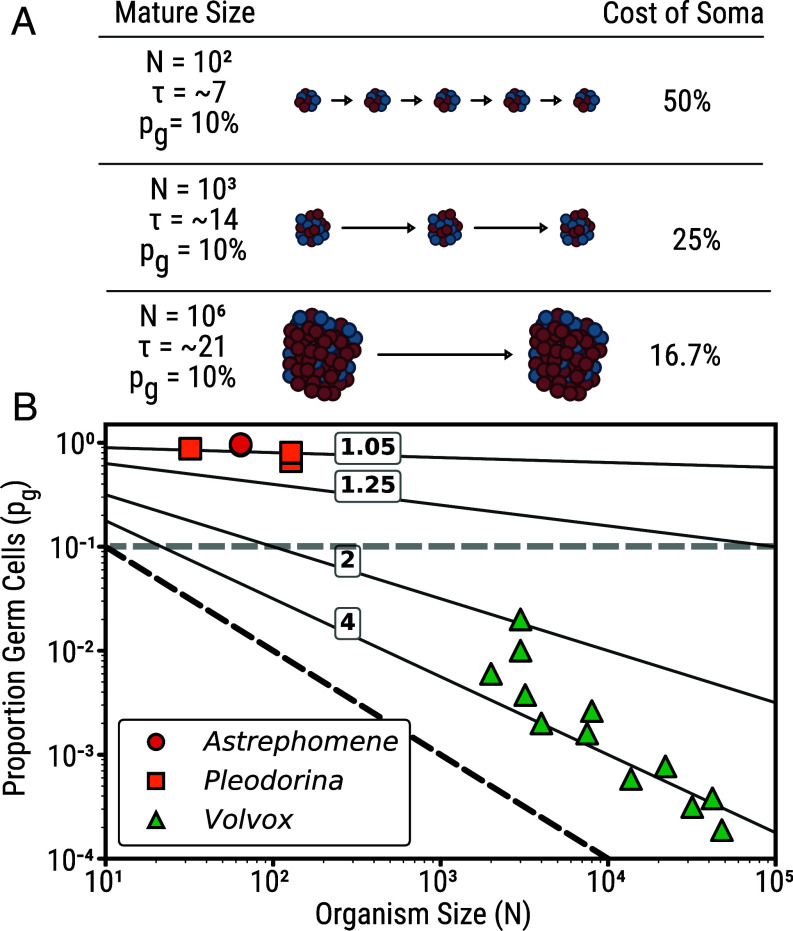
Large organism size reduces the fitness costs of somatic specialization. (*A*) Multicellular organisms develop from a unicellular propagule through synchronized cell divisions to mature size *N*, with proportion $p_{g}$ as germ cells (blue) and the remainder as somatic cells (red). Development requires $\tau =\log_{2}\left(N\right)$ cellular generations. The cost of somatic specialization (reduction in growth rate) decreases with organism size: for organisms with 10% germ cells, costs drop from 50% at $N=10^{2}$ to 16.7% at $N=10^{6}$. (*B*) Contour lines show the fold reduction in per-generation exponential growth rate for different organism sizes and germ cell proportions. The black dotted line marks the theoretical minimum of one germ cell; the gray horizontal dotted line illustrates that at a fixed $p_{g}$, larger organisms pay lower growth rate costs. Colored symbols show calculated growth-rate costs for volvocine algae species (data enumerated in *SI Appendix* and ref. 11).

Consider organisms developing from unicellular propagules through synchronized cell divisions to size *N*, with proportion $p_{g}$ as germ cells where $0<p_{g}\leq 1$. Development requires $\tau =\;\log_{2}\left(N\right)$ cellular generations. At maturity, the somatic cells die, $N\cdot p_{g}$ germ cells disperse as propagules, initiating the next generation (Fig. 2*A*). Note that this model is agnostic to the timing of germ-soma differentiation: the cost depends only on the final proportion of germ cells at maturity, not on whether differentiation occurs early or late in development, so long as somatic cells continue to divide during development. The number of organisms after *n* generations (time nτ) equals ${\left(N\cdot p_{g}\right)}^{n}$. For continuous time $P\left(t\right)=P\left(0\right){\left(N\cdot p_{g}\right)}^{t/\tau }$, where P(0) is the initial population size. We consider exponentially growing populations both for mathematical simplicity, and because it represents the most costly scenario for nonreproductive somatic cells (i.e., during periods of no growth, there is no effect of somatic cells on an organism’s fecundity).

The intrinsic growth rate *r* satisfies $P\left(t\right)=P\left(0\right)\cdot e^{\mathrm{rt}}$. Solving yields:

[1] $$\begin{array}{c} r=\frac{\ln(N\cdot p_{g})}{\tau }=\;\ln\left(2\right)\cdot \frac{\ln\left(N\right)+\ln\left(p_{g}\right)}{\ln(N)}. \end{array}$$

Maximum population growth rate occurs when all cells are germ cells ($p_{g}=1$), giving $r_{\max}=\;\ln\left(2\right)$ independent of organism size. The cost of somatic specialization equals:

[2] $$\begin{array}{c} 1-\frac{r}{r_{\max}}=\frac{-\ln(p_{g})}{\ln(N)}. \end{array}$$

This demonstrates a key insight: somatic specialization becomes progressively less expensive as organisms grow larger. Suppose an organism allocates 90% of its cells toward soma, so that $p_{g}=0.1$. The cost to the population growth rate of this somatic specialization is 50% in a 100-cell organism, but drops to 25% in a 10,000-cell organism and only 16.7% in a million-cell organism (Fig. 2*A*).

This size-tempering effect arises because development time scales as log(*N*) while reproductive losses are a fixed proportion of total cell number. Small organisms complete development in few cell divisions, causing reproductive losses to compound frequently, whereas larger organisms dilute these costs across more biomass doublings per reproductive cycle.

Having established that the fitness costs of reproductive specialization scale inversely with organism size, we now examine how germ-soma specialization evolves when somatic cells provide a compensating benefit. The specific benefits vary across taxa, from enhanced locomotion to improved survival. We explore the general principle through an illustrative model in which somatic cells increase survival of groups in response to repeated episodes of some stressor. We assume these episodes occur every $t_{s}$ time units so that within a given time *t* there are $t/t_{s}$ events. Only a fraction of groups survive each event, with survival depending on the group’s investment in soma, $1-p_{g}$. We define this survival function $s(p_{g})$ so that investment in soma provides a linear increase in survival:

[3] $$\begin{array}{c} s\left(p_{g}\right)=s_{g}p_{g}+s_{s}\left(1-p_{g}\right), \end{array}$$

where $s_{g}$ and $s_{s}$ represent the survival fraction of a group of only germ or soma cells, respectively. $s_{g}<s_{s}$ is assumed because somatic cells increase survival. Eq. **3** can be rewritten as $s\left(p_{g}\right)=s_{s}+\left(s_{g}-s_{s}\right)p_{g}$ to highlight the linear dependence of group survival on germ investment.

We can incorporate this survival function into the equation describing the population dynamics of groups:

[4] $$\begin{array}{c} P\left(t\right)=P\left(0\right){\left(Np_{g}\right)}^{t/\tau }s{\left(p_{g}\right)}^{t/t_{s}}. \end{array}$$

Using this equation, we can determine the investment in germ/soma that maximizes the population growth rate. We take the derivative with respect to germ investment $p_{g}$ and find that the optimal fitness occurs when $p_{g}^{*}=\frac{s_{s}/\left(s_{s}-s_{g}\right)}{1+\log_{2}\left(N\right)/t_{s}}$. From this equation, we find that there is a critical time ($t_{s}^{*}=\left(s_{s}/s_{g}-1\right)\log_{2}\left(N\right)$) for stress events such that when $t_{s}<t_{s}^{*}$ groups that invest in soma are fitter than those that only invest in germ cells (additional calculations in *SI Appendix*, *Supplementary Methods*). Interestingly, this critical time depends on the size of groups so that larger groups require less frequent events to select for specialization. Thus, for any frequency of stress events there is some group size above which groups increase their fitness by producing somatic cells.

This simple model also reveals a positive feedback dynamic. Once groups invest in soma ($p_{g}<1$), increasing in size leads to increased fitness. This occurs even in the absence of stress events because the population equation $P\left(t\right)=P\left(0\right){\left(Np_{g}\right)}^{t/\tau }$ has a positive derivative for $\frac{\mathrm{dP}}{\mathrm{dN}}$ when $p_{g}<1$. Indeed, there was nothing about the chosen survival function $s(p_{g})$ that favors larger groups. Rather, the fitness advantage favoring larger groups emerges because smaller groups have shorter life cycles. The cost to population growth rate of investing in somatic cells is then not only paid more often over some time period, it compounds geometrically. As groups evolve to be larger the optimal investment in somatic cells also increases, because the optimal $p_{g}^{*}∼1/\log_{2}\left(N\right)$ for large *N*. This feedback dynamic predicts a positive correlation between organism size and investment in cellular specialization.

The volvocine green algae offer qualitative support for these predictions. In this group, larger organisms have evolved progressively greater somatic investment (Fig. 2*B*). We quantified this pattern using linear regression on log-transformed species-level data (maximum reported cell number and gonidia count per species). Across 18 volvocine species with germ-soma differentiation, the proportion of germ cells scales strongly with log (cell number) (phylogenetic independent contrasts: $p_{g}\propto N^{-1.2}$, $r^{2}=0.95$, $P<10^{-10}$). This relationship holds within the genus *Volvox* alone (phylogenetic independent contrasts: $p_{g}\propto N^{-1.14}$, $r^{2}=0.86$, $P<10^{-4}$, n=12 species), spanning organisms from 2,000 to nearly 50,000 cells. Notably, this empirical scaling does not directly test the cost equation in isolation, because $p_{g}$ covaries with *N* across species. Instead, the data reflect the realized evolutionary outcome: as species have evolved different sizes, they have adjusted their somatic investment accordingly. The observed relationship $p_{g}\propto N^{-1.2}$ implies that the proportional cost to growth rate is roughly constant across species, consistent with a scenario in which larger organisms have leveraged their reduced costs to evolve greater cellular specialization rather than simply growing faster. We can roughly test the model’s quantitative predictions for one species: *Volvox aureus*, with approximately 2,040 somatic cells and eight germ cells (7, 8), is predicted to grow 3.7-fold slower than if every cell were a germ cell, similar to the observed three- to fourfold difference in growth rates between *V. aureus* and its unicellular relative *Chlamydomonas reinhardtii* (9, 10). Taken together, these patterns are consistent with the positive feedback dynamic predicted by our cost–benefit model, in which larger organisms can afford greater somatic investment, which in turn favors further increases in size.

Most work on the evolution of reproductive specialization has focused on the benefits of division of labor. Our results complement this research by quantifying the costs that these benefits must overcome, and showing that these costs depend critically on organism size. By reducing the costs of specialization, larger size removes a key constraint on reproductive division of labor during evolutionary transitions in individuality.

## Materials and Methods

Full model derivations, phylogenetic independent contrasts analysis, and species-level data on volvocine algae are provided in *SI Appendix*.

## Supplementary Material

Appendix 01 (PDF)

Dataset S01 (CSV)

## Data Availability

All data and code is publicly available at GitHub (https://github.com/Sacrozhangt/Size-and-Somatic-Specialization (11). All other data are included in the manuscript and/or supporting information.
