# Supplementary material for: The fitness costs of reproductive specialization scale inversely with organismal size
Source: Proc Natl Acad Sci U S A. 2026 Apr 6;123(15):e2536055123. doi: 10.1073/pnas.2536055123 (PMC13079367; doi:10.1073/pnas.2536055123)
Supplement: Supplementary file 1 — Appendix 01 (PDF) [file pnas.2536055123.sapp.pdf]

Supporting Information for

The fitness costs of reproductive specialization scale inversely with organismal size

Christopher Zhang, Eric Libby, Anthony Burnetti, Matthew Herron, William Ratcliff

Corresponding Author name. William Ratcliff
E-mail: ratcliffgatech.edu

This PDF file includes:

Supporting text
SI References

Supporting Information Text

Derivation of optimal germ-soma allocation with repeated stress events.

In the main text, we presented a model in which somatic cells provide a survival benefit to multicellular groups facing periodic environmental stress. Here we derive the key results: the optimal proportion of germ cells that maximizes fitness, and the critical frequency of stress events required for somatic specialization to be favored over purely germline investment.

We describe the population dynamics of groups as:

$$P(t) = P(0)(Np_g)^{\frac{t}{\log_2(N)}} \quad [1]$$

Using this we can calculate $\frac{\partial P}{\partial N}$ to determine the group size that maximizes fitness. We find

$$\frac{\partial P}{\partial N} = P(0)(Np_g)^{\frac{t}{\log_2(N)}} \left(\frac{-\ln(p_g)\ln(2^t)}{N(\ln(N))^2} \right) \quad [2]$$

We note that if $p_g = 1$ then the equation has 0 derivative. This mean that fitness cannot be improved by changing group size, since all cells are germ and there is no inherent advantage for groups being big or small. However, if $p_g < 1$ then the derivative is always positive (note $-\log(p_g)$ is positive) so that fitness always increases by increasing N. If we consider somatic cells that increase survival then the population dynamic equation becomes

$$P(t) = P(0)(Np_g)^{\frac{t}{\log_2(N)}}(s(p_g))^{\frac{t}{t_s}} \quad [3]$$

We can determine the optimal fitness as a function of p_g by taking the derivative with respect to p_g. For simplicity we rewrite the population dynamic equation as:

$$P(t) = P(0)e^{t\left(\frac{\ln(Np_g)}{\log_2(N)} + \frac{1}{t_s}\ln(s(p_g))\right)} = P(0)e^{\lambda t}, \quad [4]$$

where

$$\lambda = \left(\frac{\ln(Np_g)}{\log_2(N)} + \frac{1}{t_s}\ln(s(p_g))\right). \quad [5]$$

In this formulation, the p_g that maximizes population size $P(t)$ is the same that maximizes the population growth rate λ. We can find the optimal investment in germ by considering the derivative $\frac{\partial \lambda}{\partial p_g}$.

$$\frac{\partial \lambda}{\partial p_g} = \frac{1}{p_g \log_2 N} + \left(\frac{1}{s(p_g)t_s}\right)\left(\frac{\partial s}{\partial p_g}\right) \quad [6]$$

The derivative in Equation 6 is composed of two terms. The first term $\frac{1}{p_g \log_2 N}$ is always positive. The sign of the second term $\left(\frac{1}{s(p_g)t_s}\right)\left(\frac{\partial s}{\partial p_g}\right)$ depends on $s(p_g)$. If we assume that survival always increases with more somatic cells then this means that the derivative of $s(p_g)$ with respect to p_g, i.e., $\frac{\partial s}{\partial p_g}$, is always negative. Thus, the second term is always negative. The differences in signs between the two terms means there is a potential place where the derivative is 0, possibly a local maximum.

We note that as p_g gets very small, close to 0, the positive term grows extremely large while the second term, the negative one, does not. This means that $\frac{\partial \lambda}{\partial p_g}$ will be positive. Thus, if the derivative changes signs at some point and becomes negative then this will be a local maximum point. If this point occurs before $p_g = 1$ then it means there is an investment in somatic cells that maximizes population fitness (or equivalently growth rate). If this maximum occurs after $p_g > 1$, it means that only investing in germ maximizes fitness.

We can set this derivative to zero to find critical values where p_g maximizes population fitness.

$$p_g = -\frac{s(p_g)t_s}{\frac{\partial s}{\partial p_g}\log_2 N} \quad [7]$$

The existence of critical value(s) depend on the specific form of the survival function. In the main text we consider the simple case of the a linear survival function: $s(p_g) = p_g s_g + (1 - p_g)s_s$. If we plug this into Equation 7, solve for p_g we find:

$$p_g = \frac{s_s/(s_s - s_g)}{1 + \log_2(N)/t_s}. \quad [8]$$

We can find the critical time for stress events t_s by solving this equation for where $p_g < 1$.

There are many other possibilities for the survival function. One interesting alternative is:

$$s(p_g) = e^{\left(-p_g - \frac{1}{10} + \frac{1}{10}\cos(2\pi p_g)\right)}. \quad [9]$$

This is a monotonically decreasing function for p_g values between 0 and 1. If we solve for critical points, we can find multiple local maximum points. For example if $t_s = 5$ and $N = 10^5$, we can the derivative of Equation 6 is 0 around $p_g = .19$ (local maximum), $p_g = .67$ (local minimum), and $p_g = .78$ (local maximum). The point $p_g = .19$ happens to have the higher total population growth rate, i.e. it is the maximum for p_g between 0 and 1. This demonstrates the possibility of more complicated trajectories and outcomes then the linear case presented in the main text.

Volvocine algae data collection.

We compiled species-level data on cell number and gonidia quantity from published literature on volvocine algae. For each species, we recorded the range of reported total cell number and range of number of gonidia per organism from morphological studies. The proportion of germ cells (p_g) in Figure 2C was calculated as the ratio of maximum gonidia to maximum total cells. Because volvocine species exhibit variation in cell number, we used maximum reported values to provide a consistent measure of physiological capacity across species. Data on volvocine algae cell numbers and germ cell allocation were compiled from the following sources and are enumerated in supplemental dataset 1.

Primary literature used to compile supplemental dataset 1.

1. JR Stein, A Morphological Study of Astrephomene Gubernaculifera and Volvulina Steinii. *Am. J. Bot.* **45**, 388–397 (1958) _eprint: https://bsapubs.onlinelibrary.wiley.com/doi/pdf/10.1002/j.1537-2197.1958.tb13142.x.
2. H Nozaki, Morphology and taxonomy of two species of astrephomene in japan. *J. Jpn. Bot.* **58**, 345–352 (1983).
3. H Nozaki, H Kuroiwa, T Mita, T Kuroiwa, Pleodorina japonica sp. nov. (Volvocales, Chlorophyta) with bacteria-like endosymbionts. *Phycologia* (1989) Publisher: Taylor & Francis.
4. MOP Iyengar, TV Desikachary, *Volvocales.* (Indian Council of Agricultural Research, New Delhi), p. 532 (1981).
5. H Nozaki, Notes on microalgae in japan (10). eudorina illinoisensis (chlorophyta, volvocales). *Jpn. J. Phycol.* **34**, 143–144 (1986).
6. GM Smith, A comparative study of the species of *Volvox. Transactions Am. Microsc. Soc.* **63**, 265–310 (1944).
7. H Nozaki, Morphology, sexual reproduction and taxonomy of Volvox carteri f. kawasakiensis f. nov. (Chlorophyta) from Japan. *Phycologia* (1988) Publisher: Taylor & Francis.
8. G Kochert, Differentiation of reproductive cells in Volvox carteri. *The J. Protozool.* **15**, 438–452 (1968).
9. M Pocock, Volvox and associated algae from kimberley. *Annals South Afr. Mus.* **16**, 473–521 (1933).

Phylogenetic correction on comparative analysis.

To account for shared evolutionary history among volvocine species, we performed a phylogenetic independent contrasts (PIC) analysis on the relationship between organism size and germ cell allocation. We used a phylogeny of 18 volvocine species with germ-soma differentiation, derived from Lindsey et al. (1), with branch lengths set to one. Maximum cell number was log-transformed prior to analysis to account for the multiplicative scaling of organism size. Independent contrasts were computed for both log-transformed maximum cell number and the proportion of germ cells (p_g) using the `pic` function in the R package `ape`.

1. CR Lindsey, F Rosenzweig, MD Herron, Phylotranscriptomics points to multiple independent origins of multicellularity and cellular differentiation in the volvocine algae. *BMC Biol.* **19**, 182 (2021).
